# Targeting Dynamical Binding Processes in the Design of Non-Antibiotic Anti-Adhesives by Molecular Simulation—The Example of FimH

**DOI:** 10.3390/molecules23071641

**Published:** 2018-07-05

**Authors:** Eva-Maria Krammer, Jerome de Ruyck, Goedele Roos, Julie Bouckaert, Marc F. Lensink

**Affiliations:** Unite de Glycobiologie Structurale et Fonctionnelle, UMR 8576 of the Centre National de la Recherche Scientifique and the University of Lille, 50 Avenue de Halley, 59658 Villeneuve d’Ascq, France; jerome.de-ruyck@univ-lille.fr (J.d.R.); goedele.roos@univ-lille.fr (G.R.) julie.bouckaert@univ-lille1.fr (J.B.)

**Keywords:** adhesion, FimH, rational drug design, molecular dynamics, molecular docking, ligand binding

## Abstract

Located at the tip of type I fimbria of *Escherichia coli*, the bacterial adhesin FimH is responsible for the attachment of the bacteria to the (human) host by specifically binding to highly-mannosylated glycoproteins located on the exterior of the host cell wall. Adhesion represents a necessary early step in bacterial infection and specific inhibition of this process represents a valuable alternative pathway to antibiotic treatments, as such anti-adhesive drugs are non-intrusive and are therefore unlikely to induce bacterial resistance. The currently available anti-adhesives with the highest affinities for FimH still feature affinities in the nanomolar range. A prerequisite to develop higher-affinity FimH inhibitors is a molecular understanding of the FimH-inhibitor complex formation. The latest insights in the formation process are achieved by combining several molecular simulation and traditional experimental techniques. This review summarizes how molecular simulation contributed to the current knowledge of the molecular function of FimH and the importance of dynamics in the inhibitor binding process, and highlights the importance of the incorporation of dynamical aspects in (future) drug-design studies.

## 1. Introduction

Although commensal *Escherichia coli* bacteria live in symbiosis with their human hosts as part of the gut flora, several *E. coli* strains are pathogenic to humans [1]. These pathogens are at the origin of a wide variety of diseases including intestinal (enteritis, and diarrhea) and extra-intestinal diseases (urinary tract infections (UTIs), sepsis, and meningitis). Uropathogenic *E. coli* (UPEC) for example are the primary cause of a large majority of UTIs (up to 70–95% of community acquired UTIs) [2,3]. UTIs are often recurrent or relapsing and although they are common infections they cause serious morbidity [4] and account for substantial medical costs worldwide [2]. The standard treatment for uncomplicated UTIs is a short course of antibiotics, which are highly effective against sensitive UPECs. However, antibiotic-resistant UPEC strains are on the rise as evidenced in urine cultures of UTI patients [5,6,7] and highlighted in 2016 by the first case of an US UTI patient carrying a pan-drug resistant *E. coli* strain [8]. The emergence of multi- and pan-drug resistance bacteria as well as the latency in the development of new antibiotics highlight the need for new non-antibiotic treatment alternatives against UPEC and other pathogenic *E. coli* infections [9,10]. A promising target for such a drug development is the FimH adhesin [11,12]. Drugs targeting FimH are unlikely to induce bacterial resistance as they do not interfere with the bacterial metabolism. Furthermore, it has been shown in mice and primate studies that vaccination with FimH leads to protection against bacterial infection [13].

FimH is located at the tip of the *E. coli* type I fimbria and used by the bacteria to adhere to their host cells. Extensive research performed on murine cystitis models evidenced that type 1 pili and FimH-mediated adhesion are essential for bacterial invasion [14,15,16,17]. UPEC (and most other *E. coli* strains) express a few hundreds of these about 1 µm-long rod-shaped organelles on their cell surface to adhere in a multivalent fashion to the superficial bladder cells. Adhesion is mediated at the molecular level by FimH binding to highly-mannosylated glycoproteins (MGP). In the case of UTIs, the primary partner for FimH adhesion is Uroplakin Ia (UPIa), a MGP present on the surface of epithelial umbrella cells of the urinary tract [18].

More recently, another class of pathogenic *E. coli* strains, the adherent and invasive *E. coli* (AIEC) strains have been evidenced to be of central importance in the development of Crohn’s disease (CD) [19,20,21]. In CD, chronic inflammation of the ileal epithelium leads to the over-expression and the display of the MGP carcinoembryonic antigen-related cell adhesion molecule 6 (CEACAM6) on epithelial cell surfaces. The adhesion of these AIEC bacteria via FimH-CEACAM6 binding leads to further bacterial invasion of the gut mucosa [21,22]. Current results show that FimH antagonists can decrease the AIEC population in-vivo [23]. An anti-adhesive mannosidic compound named EB8018 (Enterome; licensed in early 2016) treating CD is currently in the human testing phase [24]. EB8018 is a divalent compound allowing for the binding of two FimH proteins at the same time. 

The FimH proteins of UPEC and AIEC have been used in the last two decades as a target in the development of precision antimicrobial drugs [25]. Such drugs have several advantages over the more traditional antibiotic drugs: (1) they are specific for a certain type of process or bacterial species (2) they do not disturb the host microbiota and (3) they are not likely to induce bacterial resistance as they interfere with the pathogen without killing it. Most of the currently known FimH inhibitors (e.g., heptyl α-d-mannopyranoside (HM), K_D_ = 5 nM) [26] have been rationally designed on the basis of structural information obtained by X-ray crystallography [24,26,27]. A new route for drug design is to include the dynamical aspects of the binding process. This review summarizes how the inclusion of dynamical information from molecular dynamics (MD) studies as well as other molecular simulation techniques can be used to gain further insight into the interaction between the anti-adhesive compound and its receptor FimH and how this information is incorporated into rational drug design to further improve the efficiency of the anti-adhesive compounds.

## 2. The Molecular Binding Mechanism of Small Mannosidic Compounds to the FimH Binding Site

### 2.1. The FimH Mannose-Binding Site

**Figure 1:**
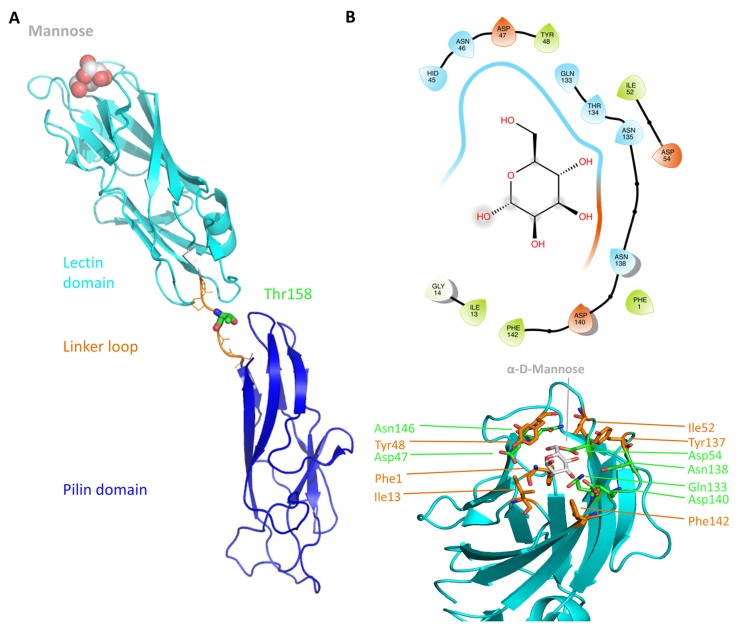
The FimH structure and organization (**A**) An elongated linker (orange) connects the pilin (blue) and the lectin (cyan) domain of FimH (PDB code 1KLF [14]). The protein is shown in cartoon and the bound α-d-mannose molecule is depicted as atom-colored (grey for carbon) van-der-Waals spheres. Additionally, the position of T158 is shown as atom-colored sticks (green for carbon). (**B**) The mannose-binding site of the FimH lectin domain. On the top the 2D diagram of the binding site is depicted (prepared with Maestro using a cutoff of 5 Å) and on the bottom the 3D representation of the same site. The mannose molecule is highlighted in gray. The polar (green) binding site residues as well as the hydrophobic rim residues (orange) are additionally depicted. The 3D protein representations in this and the following figures were prepared using Pymol [29].

The first crystal structure of an α-d-mannose molecule bound FimH was reported in 2002 [14], disclosing that FimH is composed of two structurally similar domains, both with an immunoglobulin (Ig)-like fold (11-stranded β-barrel) connected through a flexible linker (amino acids (aa.) 154–160) (see Figure 1A). The N-terminal, lectin domain (aa. 1–153) carries the mannose-binding site, whereas the C-terminal, pilin domain (aa. 161–276) mediates the connection with the other proteins of the type 1 pili. The co-crystallized α-d-mannose molecule is located in a polar pocket (see Figure 1B, Asn46, Asp47, Asp54, Gln133, Asn135, Asn138 and Asp140) of the lectin domain. Its tight binding is achieved predominantly through hydrogen (H) bonding (direct and water-mediated) and other electrostatic interactions. The binding pocket is surrounded by a collar of hydrophobic residues (see Figure 1B, Phe1, Ile13, Tyr48, Ile52, Tyr137 and Phe142).

The lectin binding site is highly specific for α-d-mannose (K_D_ = 2.3 µM) as evidenced by surface-plasmon resonance (SPR) measurements [26]. Minor changes in the chemical structure of the sugar as for example the change of the 2-hydroxyl group position (d-glucose, K_D_ = 9240 µM) or its complete removal (2-deoxy-α-d-mannose, K_D_ = 300 µM) results in compounds only very poorly recognized by FimH [26]. Only the sugar fructose, a five membered ring with the 2-hydroxyl group being axial, shows an affinity for FimH binding that is near the one of α-d-mannose, albeit 15-fold less (K_D_ = 31 µM) [26]. Most of the key residues (Phe1, Asn46, Asp47, Asp54, Gln133, Asn135, Asp140 and Phe142) shaping the FimH mannose-binding pocket are invariant throughout all known strains of *E. coli*. The mutation of any of these residues led to a loss of mannose binding and diminished virulence [14,28]. These observations are in line with the high specificity of FimH for α-d-mannose.

**Figure 2:**
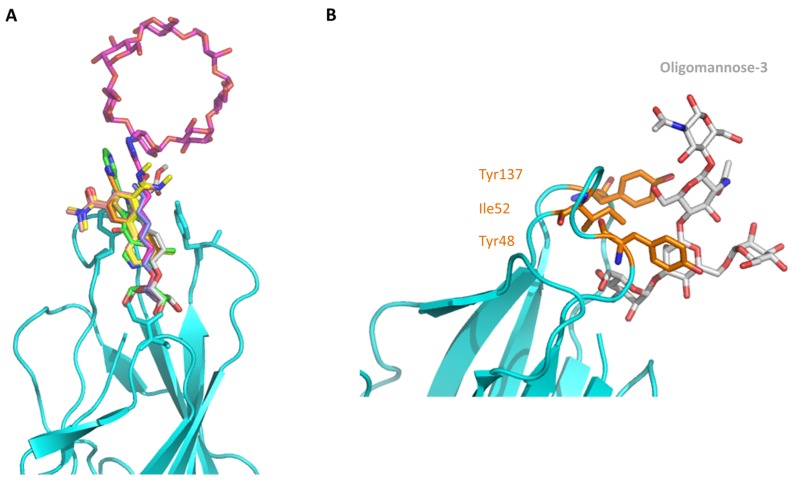
FimH in complex with different inhibitors. (**A**) The mannose ring of different recent high-affinity inhibitors is bound similarly to FimH. Shown are the following inhibitors: HM (lilac, PDB ID: 4BUQ [31]), thiazolylaminomannoside (green; PDB code 5MTS [32], β-cyclodextrin-α-d-mannoside (purple; PDB code 5AB1 [23]), para-biphenyl-2-methyl-3′,5′di-methylamide-α-d-mannoside (yellow; PDB 5F2F [33]), 8-(Methoxycarbonyl)octyl-α-d-mannoside (grey, PDB code 4AVI [34]), 3′-Chloro-4′-(α-d-mannopyranosyloxy)biphenyl-4-carbonitrile (orange, PDB code 4CST [35]), *para*-biphenyl-2-methyl-3′-methylamidemannoside (rose, PDB code 5F3F [36]). (**B**) Oligomannose-3 bound to FimH. The mannoside is highlighted in grey and the tyrosine gate residues in yellow. The FimH lectin domain is shown in cyan in the cartoon (PDB code 2VCO [37]).

As the FimH lectin domain is highly specific for mannose, and no other site was exploited so far in anti-FimH drug design, most so-far developed FimH inhibitors contain a mannose compound (see Section 3.1). In the more than 50 crystal structures of FimH in an inhibitor-bound state, that can be accessed today in the PDB database, the mannosidic moiety binds in the same way, independent of the chemical nature of the aglycon moiety (see Figure 2A). Very recently, however, a series of FimH inhibitors were designed featuring instead of a α-d-mannose ring a seven-membered ring analog (septanose rings). One among them, the 2-*n*-heptyl-1-deoxyseptanose (HS), is very promising as it features only an about 10-fold reduced affinity (K_D_ = 0.26 µM) compared to HM (K_D_ = 0.029 µM in the same isothermal titration calometry (ITC) measurement) [30]. Furthermore, the crystal structure of FimH in complex with this HS compound highlights that the septanose ring is very similarly bound as the mannose ring of HM sharing the same H bond partners (Phe1, Asp47, Gln133, Asn137 and Asp140) [30]. Further optimization of this HS might lead to a new class of potent FimH inhibitors.

### 2.2. The Tyrosine Gate and Its Impact on Mannoside Binding

The crystal structure of FimH with the branched oligomannose-3 [37] highlights the particular importance of the tyrosine gate, formed by Ile52, Tyr48 and Tyr137, for the binding of the mannose rings adjacent to the first mannose ring bound in the pocket (see Figure 2B). The tyrosine gate is located at the entry of the binding pocket and forms part of the hydrophobic collar (see Figure 1B). It is at the level of the tyrosine gate, that the isolated FimH lectin domain differentiates between different high-mannosidic glycans, mainly based on their capability to form hydrophobic interactions with this gate. A recent combined molecular simulation and experimental study highlighted the coupling of the motion of the two tyrosine residues via Ile52 (see Table 1) [38]. The tyrosine gate has attracted large interest because of its potential to generate nanomolar affinities for mannosides conjugated to hydrophobic aglycons through the formation of favorable van der Waals and stacking interactions within the gate. Based on crystallographic data, different inhibitor interaction modes have been evidenced: the non-glycon substituents either travel through the gate and interact in multiple stacking modes either (1) with Tyr48 (Tyr48-loving) or (2) with Tyr137 (Tyr137-loving), or (3) bypass the tyrosine gate and interact with either one or both the tyrosine residues from the outside [31,34,35,37,39,40,41].

The interaction of the aglycon moiety of the anti-adhesives with one or several tyrosine gate residues has been shown to impact the affinity of the inhibitor. Furthermore both Tyr48 and Tyr137 have been evidenced to be highly dynamic [34,38]. A detailed molecular understanding of the mode of action of the tyrosine gate, including its dynamical behavior, is therefore required in order to design more efficient inhibitors.

We recently generated single-residue FimH mutants in which one of the two tyrosine-gate residues was mutated to alanine (Y48A and Y137A). The effect of these mutations on the binding of three synthetic ligands (1,5-anhydro-d-mannitol, HM, and 4-biphenyl-α-d-mannose) was tested by X-ray crystallography, affinity measurements and molecular simulation studies [38]. The experimentally determined affinity data highlight the importance of Tyr137, as its mutation clearly alters the binding properties of the FimH lectin independently from the ligand used (Table 1). No major structural changes were evidenced in the mutant by X-ray crystallography and CD measurements. Only the combination of quantum mechanics (QM) calculations and MD simulations revealed why the FimH Tyr137Ala (Y137A) mutant shows such a dramatic loss of affinity without being in direct contact with its mannose ligand: in the ligand-free state of the FimH Y137A mutants, several of the binding site residues (48, 136, and 137) exhibit backbone dihedral angles that are normally only found after binding of the mannose. This is because the Y137A mutation disrupts a dynamic coupling between Tyr137 and Tyr48 via the inner Ile52 residue and holds the binding cavity in a highly energetic mannose binding conformation [38]. In addition, the ligand retained a higher flexibility in the binding site of the Y137A mutant compared to the wild-type. In contrast to this, the in-silico mutation of Y48A only minimally affects the binding affinity of the different ligands as shown by smaller observed effects on the flexibility of the ligand and on the protein local dynamics. This is in good agreement with entropy-enthalpy compensation effects seen in ITC measurements performed within our study [38] as well as in an earlier study of the Y48A mutant [49]. The latter study also showed, using NMR, X-ray crystallography and SAXS measurements that the Y48A mutation does not affect FimH structure and function. 

### 2.3. The Conformational States of FimH

**Figure 3:**
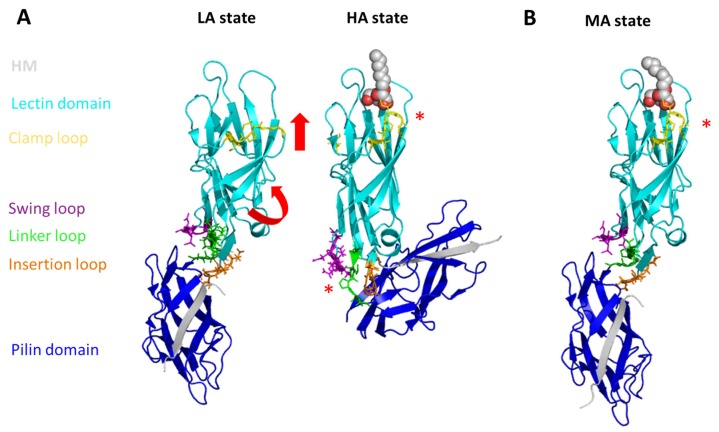
The conformational flexibility of FimH. The LA (**left**; PDB code 4XOD), HA (**middle**; PDB code 4XOB), and MA (**right**; PDB code 4XOE, chain G and H) state are depicted. Following a β-sheet twisting mechanism, the lectin domain is elongated and straightened in the HA (MA) state (red arrows) leading to a local conformational change in the mannose-binding site (red star) locking it in its high affinity conformation. In the HA state the pilin domain is elongated and the link between the pilin and the lectin domain is weakened. The lectin (cyan) and the pilin (blue) domain as well as the clamp (yellow), swing (purple), insertion (orange), and linker (green) loops are shown in cartoon (domains, loops) and in lines (loops). The co-crystallized peptide is shown in grey cartoon. The HM bound to the HA and MA state is shown in van-der-Waals spheres (grey).

All inhibitors discussed so far in this review target FimH in its high affinity (HA) state, however, FimH can also exist in a low affinity (LA) state (see Figure 3A), which is at least 100 times less efficient in mannose binding [50]. In the absence of any force FimH is in its LA state, most likely loosely adhering to its receptor, allowing thereby the UPEC or the AIEC bacteria to change their position and move along the tissue [51,52]. Shear force can be observed in the human body in the form of the flow of fluids such as mucosal secretions used as natural body defenses against bacterial colonization [53]. Furthermore, shear forces can also act on UPEC in the form of urine flow. Under laboratory conditions, force application triggers the conversion of FimH from its LA state to its HA state. The conversion most likely allows the bacteria to withstand the vigorous shear stress imposed by the (human) host. The combination of steered MD (sMD) simulations and atomic force microscopy (AFM) measurements allowed to get insight into the molecular origin of the conformational change [54,55,56]. Moreover, using the fimbrial tip (consisting of FimH, FimG followed by one FimF) structure [57], a coarse-grained lattice Boltzmann MD simulation study showed that the application of fluid flow leads to a drastic alternation of the complex conformation [58]. In these simulations, the chain stretched according to the fluid velocity drag in accordance with a shear-force dependent conformational change. In the sMD simulations of the isolated FimH tensile forces were applied between residues in the mannose-binding pocket or the mannose and residues at the end of the interdomain linker chain [54]. In the study of the fimbrial tip tensile force were applied between the binding site residues and the donor-strand of the second FimF molecule [55]. Independent of the used sMD approach the interdomain linker loop extended under the applied force. In line with the observed importance of the linker, SPR experiments highlight that natural variants of FimH with different amino acids in the position 158 (located in the linker loop region, see Figure 1A) show different responses to stress [22,46,47]: the adhesion strength of the uropathogenic UTI89 E. coli strain with a threonine at position 158 of FimH shows an optimum at higher shear, whereas in strains (AIEC7082 and LF82) carrying an alanine or a proline respectively at aa. position 158, bacterial binding is less or not enhanced with increasing fluid shear.

The crystal structure of FimH as part of the multi-protein fimbrial tip (FimH followed by one FimG and two FimF molecules, the last one stabilized by the FimC chaperone) highlight the FimH in its LA state [57]. Even in absence of the FimG proteins, the LA state can be stabilized by the co-crystallization of FimH with a DsG peptide, which fills the place of the donor-strand of FimG complementing the missing β-strand of the Ig-fold of the FimH pilin domain [59]. In the FimH LA structure (see Figure 3A), the anchoring (pilin) domain of FimH interacts with the mannose-binding (lectin) domain and causes a twist in the β-sandwich fold of the latter. This loosens the mannose-binding pocket on the opposite end of lectin domain. The HA was observed in the isolated lectin domain structures [26,37], the FimH-FimC structure [14,60], and the HM-bound FimH-DsF peptide structure [59]. In these structures the lectin domain is untwisted and elongated compared to the LA state resulting in a tight, high-affinity mannose-binding pocket. In the FimH-FimC structures the HA is most likely stabilized by the FimC chaperon that is wedged between the FimH lectin and pilin domain thereby separating the two domains. Three flexible loops, the so-called swing (aa. 27–33), linker (aa. 154–160) and insertion (aa. 112–118) loop, have been identified to mediate contact between the pilin and lecin domain in the LA state (Figure 3) [57]. During the conformational change of the LA to the HA state these loops are rearranged leading to the rupture of the inter-domain connections and elongation of the linker loop. SPR experiments with natural variants of FimH (e.g., aa. 158; Table 1) found in different *E. coli* strains corroborate the importance of the linker loop in the shear-dependent conformational change [22,46,47]. The analysis of sMD simulations combined with experimental data highlighted that below a force of about 60 pN, the unbinding of the mannose is mainly observed from the LA state (with a rate of 6 s^−1^ [61]. Above that force, the conformational change to the HA state is the main occurring event (with a rate of 0.00125 s^−1^) [55,62]. 

The mechanical activation of FimH (the switch of FimH from its LA to its HA state) was proposed to be due to allosteric coupling of its two domains: the pilin domain functions as an allosteric autoinhibitor of the lectin domain, which is pulled away by the mechanical force (as described above). The interdomain loops (see Figure 3) have been identified as structural elements important for the allosteric activation of FimH [48,54,57]. Based on computational structural analysis (using the Rosetta Design tool) [63], MD simulations, site-directed mutagenesis and enzyme-linked immunosorbent assay (ELISA), a β-bulge (aa. 59 to 63) and α-switch (aa. 64 to 71) region have been pinpointed to be also tightly coupled to the pilin domain and playing an important role in the allosteric change [64]. These regions and the clamp loop (aa. 10 to 15), which closes the binding site in response to mannose binding (see Figure 3), have also been identified as significantly changing their conformation in an extended MD study of the FimH HA/LA change using the Anton supercomputer [65]. Based on crystallographic data [57] and MD simulations [65], a large β-strand (aa. 16 to 22) connecting the clamp loop was identified as mediator to propagate the allosteric signal from the binding site to the pilin domain. Thus a possible treatment alternative to antibiotic treatment could be to develop allosteric inhibitors or antibodies against FimH [66]. Several anti-FimH antibodies have been identified so far [66,67,68]. The antibodies carry out inhibition using either an allosteric (mAb21, [66]), competitive, orthosteric (mAb475, [68]) or non-competitive, parasteric (mAb926, [67]) binding mechanism. Whereas the mAb21 was found to significantly enhance adhesion, most likely by stabilization of the HA state [58,60], the mAb926 and mAb475 antibodies are strongly inhibiting adhesion, the latter via blocking the switch from the LA to the HA state [67]. In the context of antibody design, MD simulations are a helpful method as they allow to verify if epitopes, identified from static structures, are also accessible to the antibody considering the dynamical nature of the protein [69]. 

Recently, the crystal structure of a HM-bound FimH-DsG peptide complex highlighted a third possible conformational state, named the middle affinity (MA) state [59]. In this state, the interdomain loops are in the same conformation as in the LA state, whereas the clamp loop already closed upon the mannose-binding site (see Figure 3B). MD simulations highlighted that the MA state is stabilized by ligand binding as after in silico removal of the HM led to a spontaneous relaxation of the clamp loop relaxes back to the LA state [59].

Although the conformational flexibility of FimH is the largest contributing factor in the shear-force-dependent binding strength, the other fimbrial tip proteins (FimG and FimF) also play a crucial function in the adhesion process. The quaternary structure of the multi-protein fimbrial tip was proposed to be highly flexible to optimize the binding rate [51] in contrast to the rod which was shown to be rigid [70]. Indeed both MD and NMR studies of FimG-FimF and FimF-FimF dimers highlight high levels of mobility [55,71]. The rigid rod has been shown to be able to recoil under increased force conditions to prevent breakage of the high-affinity mannose bond in the FimH lectin domain [72]. The fimbria and in particular FimH thus allow the bacteria to hold firmly onto the cells in the presence of a shear stress (such as the urine flow) as well as in the absence of this stress in order to detach and change location. The phenomena of sustained FimH binding (slower off rates) under stress conditions was descripted by “catch bonds” [73]. Catch bonds were also observed in other adhesive proteins and are thus likely to be a common phenomenon for proteins involved in various adhesion processes [61,74]. 

## 3. Rational Drug Design of FimH Inhibitors

### 3.1. Monovalent FimH Inhibitors Targeting the Mannose-Binding Pocket of the HA FimH State

As historically only the HA state of FimH was available, several classes of mannosidic inhibitors have been rationally designed based on structural information targeting the FimH mannose-binding site of the HA state [42,75]. These compounds can be subdivided into the following chemotypes: alkyl/aryl mannosides, biaryl mannosides, mannose ring modifications including *O*-glycosidic bond replacement leading to *N*-, *S*-, or *C*-linked compounds (Table 2) [24,39]. Several of these compounds have been crystallized in complex with FimH recently, highlighting the fact that the mannoside moiety binds in the same fashion, independently of the identity of the atom type at the glycosidic bond [36,40,76,77,78] (Figure 1C) and that the non-sugar moiety interacts with the tyrosine gate in one of the three modes described in Section 2.2. The added advantage of non-*O*-glycosidic linked compounds is that they are less sensible to host glycosidases and thus might be better suited for therapeutic use [24]. An extensive overview of all physiochemical properties of the currently known FimH inhibitors has been published elsewhere (see for example Reference [24]). Of particular interest are the recently developed thiazolylaminomannosides (TazMans), as they have been identified as potent anti-adhesives of different *E. coli* strains isolated from patients with CD, cystitis or osteoarticular infections [77,78,79].

As the search for new FimH inhibitors is largely structure-driven, several examples exist in which structural and affinity data were combined with molecular docking in a rational drug design approach [27,36,80,81]. In 2006, shortly after the first X-ray structures of FimH became accessible, a first combined experimental/molecular docking study was published [80]. This study highlights that for most of the tested compounds (alkyl and squaric glycans) the computed docking score is related to the affinity data obtained by ELISA measurements. The combination of docking with bioassays allowed to determine the binding mode of squaric acid monoamine mannosidic compounds to FimH and their affinities [82]. Based on docking poses with biphenyl derivatives with nanomolar affinities for FimH, new biphenyl inhibitors were designed, some of which showed higher affinities, increased solubility and slightly improved pharmacokinetic properties as the original compounds [81]. In 2013, over 100 mannoside compounds were also used to develop a multi-dimensional quantitative structure-activity relationship (mQSAR) and to develop an automatized tool box for in-silico rational drug design and MD simulation [83]. Docking models of *C*-linked mannosides in *R*- and *S*-isomer highlighted that the binding of the *R*-isomer to FimH is energetically favored due to a water-mediated H-bond with Asn138 and Asp140, which is only observed in the former [36]. The induced-fit docking of *C*-, *O*-, *N*- and *S*-glycosidic compounds to FimH further indicated that the position of this water and thus the distance to the linkage depends on the identity of the exocyclic atom (distance water *O*-exocyclic atom 2.9 Å for O and N, 3.5 Å for S and 3.6 Å for C) [27]. The dependence of the distance of the water to the glycosidic linkage was also highlighted in a crystallographic study of *C*-, *N*- and *O*-linked compounds [40]. The position of the water in the different bound states might influence the affinity of FimH for the different compounds.

**Figure 4:**
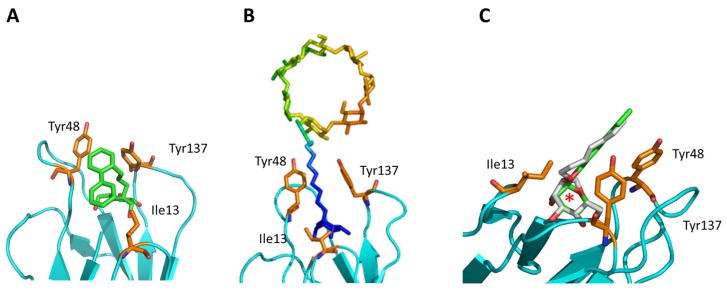
Recent results including the dynamics aspect of the FimH-ligand complex (formation). (**A**) A minor MD conformation (populated with 11%) shows the C117 second phenyl ring orientated towards C117 [76]. (**B**) The position of bCD in the binding pocket (PDB code: 5AB1). The ligand is colored according to the structure factor (from blue: rigid to red: highly flexible). (**C**) Comparison of the septanoside position (green; HS; PDB code: 5CGB) compared to a mannoside compound (grey; HM; PDB code: 4BUQ). The difference in the sugar ring is highlighted by a red asterisk.

Only a few examples exist in which the flexibility of the ligand and the very dynamical behavior of the protein were taken into account into the drug development and/or the understanding of the underlying mechanism(s). One such example is the determination of an alternative binding position of a *C*-linked *ortho*-subsituted biphenyl mannose derivative (C117) in the FimH binding site in its HA state [39]. After overlaying the sugar of the NMR-solution C117 with the position of the mannose ring of other FimH inhibitors in the binding site indicated that the C117 first phenyl moiety of C117 interacts with Tyr48 and the second one points towards Ile13 (Table 1), which is part of the clamp loop (see Figure 2). In the absence of structural information of the C117-FimH complex, the existence of such a secondary binding position for C117 could only be evidenced by combining molecular docking and MD simulations. Indeed, following the dynamics of the generated C117-FimH complex the Ile13-oriented binding mode could be identified as a minor binding mode (see Figure 4A) [76]. The Ile13-oriented binding mode was also identified as the secondary binding mode for biantennary mannosides using molecular docking [44]. The identified minor binding mode is of particular interest as the clamp loop (see Section 2.3) undergoes a major conformational change when FimH forms high-affinity catch bonds with mannosides [76] and changes from the LA to the MA and eventually to the HA state. Mannosides targeting and stabilizing this secondary binding site are in the focus of further inhibitor development as they might alter the kinetics of the FimH conformational change.

Another example of how molecular simulation can contribute to understand the drug properties is captured in the case of a β-cyclodextrin (bCD)-containing HM (bCD-1HM; Figure 4B). This inhibitor was recently shown to disrupt the attachment of *E. coli* to the bladder or gut of mice models of cystitis and CD, respectively [84,85]. The crystal structure of the bCD-1HM HA FimH complex highlighted that the HM part of this compound adopts a similar conformation as the isolated HM antagonist in the FimH binding site [23]. According to the obtained electron density and MD simulations, the bCD moiety does not form any significant interactions with the protein and moves freely in solution. Thus, it does not seem to impact bCD-1HM binding. Surprisingly however, bCD-1HM has a much lower effect as HM on the capability of *E. coli* LF82 strains to adhere to intestinal epithelial cells (T84) [23]. MD simulations of the bCD-1HM compound alone in water allowed to provide a possible explanation of the observed effect difference: the bCD moiety of bCD-1HM seems to fold back and interact with the HM moiety of the compound thereby locking it in a state unfavorable to FimH binding as the mannose part is shielded by the interaction. The addition of the bCD moiety to HM is thus likely to modulate the pharmacokinetics of the compound but not the in-vivo affinity.

Metadynamics simulations of the inhibitors alone in solution also allowed to understand why the change of the mannose sugar to a septanose ring led to an increased entropic penalty in ITC measurements even so they show similar binding to FimH (Figure 4C) [30]: the HM (with a mannose) ring had only a single energy minimum for the considered O1–C1–C4 angle and the O1–C1–O5–C5 of dihedral torsion, which represents the HM conformation in the FimH bound state, whereas for the corresponding septanose (HS) compound a more shallow energy landscape was observed with two energy minima for the corresponding angle/dihedral, one of which does not correspond to the bound state of the compound. Thus, the binding of the septanose derivative leads to a higher reduction in conformational flexibility of the sugar and thus accounts for the higher entropic cost.

As shown in the examples, the incorporation of data on the dynamics behavior of the protein, the complex and the ligand in water, often originating from MD simulations, allows to describe and therefore understand the dynamics of the binding process. It thus complements the more traditional approaches such as crystallography and affinity measurements from which static pictures are obtained. 

### 3.2. Multivalent FimH Inhibitors Targeting the Mannose-Binding Pocket

Adhesion of pathogenic E. coli is mediated by multiple type 1 fimbria and thus FimH binding to MGP displayed on the host cells (for example UPIa in the case of UPEC [18] or CEACAM6 in the case of AIEC [86]). It is well known that so-called glycoclusters can improve the affinity for lectins to a large extent [11,87]. Moreover, rather high concentrations of HM are needed to obtain 90% reduction of the bacterial load in a mouse model [42,85], making the design of better binding inhibitors needed. Therefore, multivalent antagonists were designed mimicking the clusters of glycans on the host cells [11,12]. These multivalent versions have the advantage over the monovalent counterpart that they could induce FimH aggregation leading to fimbrial entanglement followed by the formation of large bacterial aggregates that are less prone to adhering to human epithelial host cells. For example, a multivalent version of bCD-1HM carrying seven HM on the bCD ring (bCD-7HM) was shown to interact with different FimH molecules simultaneously and to induce FimH aggregation and precipitation [85,88]. Interestingly bCD-7HM, highlighted a 100-fold reduction in the effective dose in CEACAM6-expressing mice compared to its monovalent version [84]. Further development of more efficient multivalent inhibitors will largely benefit from the incorporation of the dynamical behavior, as assessable by MD simulations, of the complex and the inhibitor alone in water.

### 3.3. Alternative Binding Positions for Inhibitors

**Figure 5:**
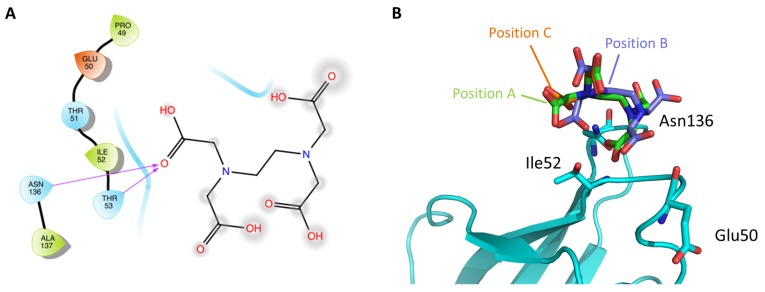
EDTA-binding site in the FimH lectin domain. (**A**) The 2D diagram of the binding site is depicted (prepared with maestro using a cutoff of 5 Å) (**B**) on the right the location of the binding site in the crystal structure (PDB code: 5FX3 [38]) is shown. Slightly different, alternative positions (**A**–**C**) of EDTA have been observed in the crystal structure and are colored differently (green, lilac, and orange).

An alternative route to develop higher affinity FimH antagonists would be to target other off-site positions instead of improving the binding affinity of FimH inhibitors that target the mannose-binding pocket. Targeting such off-site binding positions might have the advantage that such an inhibitor might block the protein in a state non-accessible to MGP binding. Recently a promising off-site binding pocket has been serendipitously discovered in the ligand-free Y137A FimH mutant crystal structure [38]. In this structure a single ethylenediaminetetraacetic acid (EDTA) molecule was observed to be bound in several orientations near to Glu50, Thr53, and Asn136 (see Figure 5). 

Most of these residues have been shown to be important for the shear-force enhanced *E.coli* adhesion to vascular epithelium cells (Table 1) [45]. The EDTA molecule was not found in the mannoside-occupied wild-type, Y48A or Y137A mutant FimH structures in spite of identical extraction, purification and crystallization protocols [38]. The relaxation of the FimH mannose-binding pocket due to the Y137A mutation (see Section 2.2) might have allowed for the binding of EDTA. The newly discovered EDTA-binding site is close to a belt of positively charged residues (aa: Arg60, Arg92, and Arg132), which are moreover strictly conserved within *E. coli.* This might indicate a protein-docking pocket in the continuation of the mannose-binding site of FimH [27]. Investigation of the flexibility of the bound EDTA as well as the design of specific compounds targeting this site will allow in the future to judge the importance of this site in the FimH adhesion process.

Also the allosteric inhibition of FimH (see Section 2.3) by side-specific antibodies could be an alternative route for treatment of *E. coli* infections such as UTI. Promisingly, a vaccination study with FimH highlighted protection against bacterial infection in the case of mice and primates [13] and antibodies inhibiting the conformational change from the LA to the HA state have been described [67]. In the design of more efficient antibodies, the incorporation of dynamical data, such as provided by MD, will prove helpful. In more general terms, the investigation of the conformational flexibility of the FimH protein and its different affinity states might open new avenues for non-mannose-binding site inhibitors of FimH.

## 4. Molecular Simulation as a Tool to Study FimH Function and Inhibition

A wide variety of different molecular simulation techniques as protein-ligand docking, MD and QM calculations were applied to FimH in order to study its conformational change, the binding of its substrates and inhibitors and to design new and more efficient anti-adhesive molecules targeting FimH. The different methods range from the investigation of model systems consisting of a few atoms like in QM, to a few thousands of atoms representing the entire protein as in molecular docking or to several tens of thousands of atoms, describing the solvated protein in an explicit solvation sphere in MD simulations. These methods representing both a static (as in QM and docking) and a dynamic modelling (as in MD) were applied together with different experimental techniques including SPR, ITC, X-ray crystallography or NMR.

All different molecular simulation approaches have their own advantages and problems. Force-field based methods such as MD for example are computationally very efficient and can handle large systems, giving an (almost) correct representation of the biological reality. In contrast, QM calculations are very time consuming and can handle only a fraction of the real system. However, force-field based methods suffer from serious shortcomings in e.g., the description of charge transfer, halogen bonding and polarizability (see for example [89] and references herein). As such, it is expected that QM calculations outperform the force-field based approaches in accuracy of (relative) energies (see for example [90] and references herein). When used for specific purposes, QM calculations on small models can thus add to the classic (MD) description. An example of a combined QM and MD approach is the study of the Y48A and Y137A mutation impact on FimH inhibitor binding [38] (see Section 2.2).

In most studies on FimH, molecular simulation was used to understand functional details of the protein not decipherable by the *priori* performed experiments. Example are the study of the molecular origin of the increased entropic penalty in ITC measurements if the HM mannose sugar is replaced by a septanose leading to HS [30] or the determination of the molecular reason for the Y137A mutation effect on the FimH HM affinity [38]. However, recent studies use molecular simulation as tool to predict effects on FimH function and regulation, which are afterwards proven experimentally. Examples hereof are the generation of a recombinant fusion proteins as a possible UTI vaccine [91,92]: the three-dimensional structural models of FimH fused to either flagellin [91] or MrpH [92] produced using molecular modelling were tested in-silico for their binding affinity towards Toll-like receptors. The fusion proteins with the best binding affinities also showed immune responses in an cell-based assay [92] and in mice experiments [91]. A similar combined molecular simulation experimental approach was followed to generate dimeric and trimeric fusion proteins of FimH with CsgA, and PapG adhesins [93].

It is a necessity for the development of UTI or CD vaccines as well as for the development of treatment alternatives to antibiotics for these diseases to combine experimental and theoretical approaches in future research. Molecular simulation can help to predict possible conformations and interactions as well as affinities of ligands and can thus explain experimental results and/or allow for the design of new experiments.

## 5. Conclusions and Outlook

The use of molecular dynamics simulation in complement to crystallographic assays offers a powerful combination to study ligand binding. For instance, the application of molecular dynamics simulation in combination with quantum-chemical calculations have allowed to understand the molecular importance of the FimH tyrosine gate and the impact of mutation of its residues on binding affinity. The incorporation of dynamical information on the wild-type FimH lectin domain in the ligand-free and ligand-bound state into a structure-based rational drug design approach allowed for the identification of a previously unidentified and promising binding mode of the ligand in the FimH binding site, in which it is oriented towards the clamp loop. These two examples among others highlight the added value of molecular simulation in the drug design of inhibitor molecules, here targeting FimH.

Further applications of molecular simulations techniques could be the identification of alternative binding positions for anti-adhesives on the FimH lectin domain, such as the recently identified EDTA binding site, or of compounds binding tightly to these positions. Such compounds might fix FimH in an off-path conformation and could thus abolish FimH receptor binding. In a similar fashion, simulation techniques can be expected to lead to the identification of key residues in the conformational change which could be then be targeted.

Molecular simulation will allow to rationally design new anti-adhesives either being specific to a single FimH conformational state or to all states and will give further insight into the molecular details of FimH binding. Therefore, it will provide the necessary details allowing experimentalists to design and perform new and more precise experiments proving and complementing the concepts provided in-silico.

It is our expectation that molecular simulation integrated with experimental techniques will lead to new routes for drug development not only for bacterial adhesins but for a variety of proteins involved in bacterial infection. In contrast to the currently used antibiotics, precision antimicrobial drugs will allow to specifically target selected bacterial strains and will thus constitute a valuable non-antibiotic alternative treatment.

## Figures and Tables

**Table 1:** Residues from the FimH lectin domain important for its function (and discussed in this review) are listed. These residues are either (i) involved in binding of the algycon moiety in the FimH mannose-binding pocket, (ii) important for the conformational change of FimH or (iii) have been shown to be involved in promising alternative binding positions. For each residue the available experimental evidence as well as the insight gained from molecular simulation shortly summarized. The most promising residues are highlight by an asterisk. The sequence from the UPEC strain UTI89 was used.

Residue	Important Due to	Exp. Evidence	Insight from Molecular Simulation
Ile13	Located in the clamp loop (changes conformation due to shear force) Possibly involved in alternative binding position	Ile13 forms van der Waals interactions with the C1–C2 bond of mannose [42] Crystal structures of the HA and LA state highlight the movement of the clamp loop [43]	The aglycon moiety of the C117 and of biantennary mannosides orients towards Ile13 [39,44].
Glu50	Part of a possible new binding site for anti-adhesives	EDTA binding site [38] Implied in the shear-force dependent conformational change [45] Less adhesion of the E50A mutant under shear [45]	
Ile52	Belongs to the tyrosine gate	Attributed to the tyrosine gate on the basis of crystal structures [42]	Mediates coupled motion of Tyr48 and Tyr137 [38]
Thr53	Part of a possible new binding site for anti-adhesives	EDTA binding site [38] Implied in the shear-force dependent conformational change [45] Less adhesion of the T53A mutant under shear [45]	
Asn136	Part of a possible new binding site for anti-adhesives	EDTA binding site [38]	
Tyr137	Belongs to the tyrosine gate Binding of the aglycon part in the mannose-binding moiety	Y137A mutation significantly reduces FimH affinity towards f HM [38]	The flexibility of the bound HM is increased in the Y137A mutant; The apo mutant already is in a quasi-bound configuration [38]
Thr158	Implicated in the shear-force dependent conformational change	Natural variation leads to bacteria with different stress responses [22,46,47]	A force was applied to this residue in the sMD simulation [48]

**Table 2:** Classification of FimH mannosidic inhibitors. For each compound type one or more examples with their affinities are listed. PDB codes for wild-type structures of FimH in complex with the corresponding inhibitors are given. The following abbreviations are used: FDA for fluorescence polarization assay; ELLSA for Enzyme-linked lectinosorbent assay; HAI for hemagglutination inhibition.

**(A) Different *O*-Linked Mannosidic Compounds**						
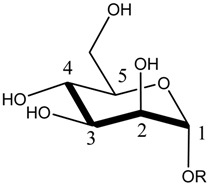						
**Compound Type**	**Example (s) (R=)**	**Measure (Technique)**	**Value [nM]**	**Ref.**	**PDB Code**	**Ref.**
Mannose	H	K_D_ (ITC)	1672.2	[34]	1KEF	[14]
		K_D_ (SPR)	2300.0	[24]		
		EC_90_ (HAI)	>1 mM	[24]		
Alkyl mannosides	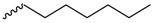	K_D_ (SPR)	5.0	[26]	4BUQ 4LOV 4XOE 4XOB	[31] [49] [59] [59]
		K_D_ (ITC)	28.9	[38]		
		K_D_ (ITC)	7.3	[34]		
		K_D_ (FDA)	28.3	[35]		
		EC_90_ (HAI)	1500.0	[24]		
		EC_90_ (HAI)	6300.0	[39]		
		IC_50_ (ELLSA)	160.0	[24]		
	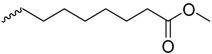	K_D_ (ITC)	23.6	[34]		
Aryl mannosides	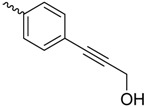	K_D_ (ITC)	18.3	[34]		
	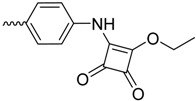	IC_50_ (Bioassay)	1730.0	[82]		
Biaryl mannosides	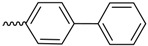	K_D_ (ITC)	17.7	[38]	5FWR	[38]
		K_D_ (FPA)	15.1	[35]		
	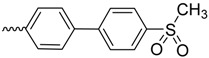	K_D_ (ITC)	3.5	[81]		
**(B) Mannose Ring Modifications**						
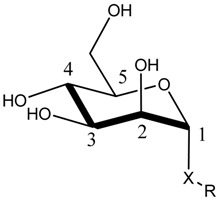						
**Ring modification**	**Example(s) (R=)**	**Measure (Technique)**	**Value [nM]**	**Ref.**	**PDB Code**	**Ref.**
*N*-linked compounds X = N	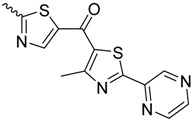	IC_50_ (ELLSA)	70.0	[32]	5MTS	[32]
	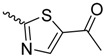	IC_50_ (ELLSA)	205.0	[32]	3LZ2	[77]
*C*-linked compounds X = C	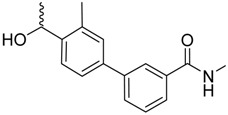	EC_90_ (HAI)	3.1	[36]		
	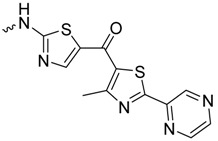	IC_50_ (ELLSA)	194.0	[32]		
*S*-linked compounds X = S	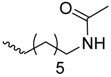	IC_50_ (ELLSA)	146.0	[23]		
